# Nontuberculous mycobacterial infection following cat scratch in the setting of topical steroid use

**DOI:** 10.7759/cureus.38901

**Published:** 2023-05-11

**Authors:** Radhika Shah, Sheevam Shah, Priscilla R Lyon, Palak Parekh, Robert Plemmons

**Affiliations:** 1 Dermatology, Baylor Scott & White Medical Center - Temple, Temple, USA; 2 Pathology, Baylor Scott & White Medical Center - Temple, Temple, USA; 3 Wound Care, Baylor Scott & White Medical Center - Temple, Temple, USA

**Keywords:** local immunosuppression, mycobacterium chelonae, cat scratch, nontuberculous mycobacterial infection, atypical mycobacteria

## Abstract

Infections due to nontuberculous mycobacteria (NTM) are caused by mycobacterial species other than *Mycobacterium tuberculosis*, *M. leprae*, and *M. bovis*. Patients who are immunocompromised have increased susceptibility to pulmonary, lymphatic, and skin infections by these pathogens. We present a case of a 78-year-old male who presented to dermatology with a left dorsolateral hand infection after sustaining cat scratches in the setting of topical steroid therapy for suspected pyoderma gangrenosum. A shave biopsy of the lesion showed granulomatous dermatitis and associated acid-fast bacilli, while tissue culture grew *Mycobacterium chelonae*. This case demonstrates cat scratches as an uncommon risk factor for cutaneous NTM disease. Although an association between cat scratches and human NTM infections has only been reported in two previous cases, it must be considered in cases of unusual and persistent cutaneous lesions, especially in immunocompromised patients, even those with only local immunosuppression from topical agents.

## Introduction

Infections due to nontuberculous mycobacteria (NTM) are caused by mycobacterial species other than *Mycobacterium tuberculosis*, *M. leprae*, and *M. bovis*. These acid-fast bacilli (AFB) can be further classified into four groups: photochromogens, scotochromogens, nonchromogens, and rapid-growers [1]. They are notably opportunistic and cause disease in those lacking adequate host immunity either by disease states, such as HIV/AIDS, or use of certain immunosuppressive medications [2]. Infection typically manifests in the form of pulmonary, lymphatic, or skin/soft tissue disease. NTM disease of the skin is relatively common but is often initially misdiagnosed [1, 3]. The most common causes of cutaneous infection include *M. marinum*, *M. abscessus*, *M. fortuitum*, and *M. chelonae* [4]. The true incidence of NTM infections is difficult to determine since the disease is not communicable and may be under-reported, but the incidence appears to be increasing. In a South Korean epidemiologic study conducted from 2007 to 2016, it was found that incidence more than tripled from 6 cases per 100,000 people in 2008 to 19 cases per 100,000 people in 2016 [5].

## Case presentation

**Figure 1:**
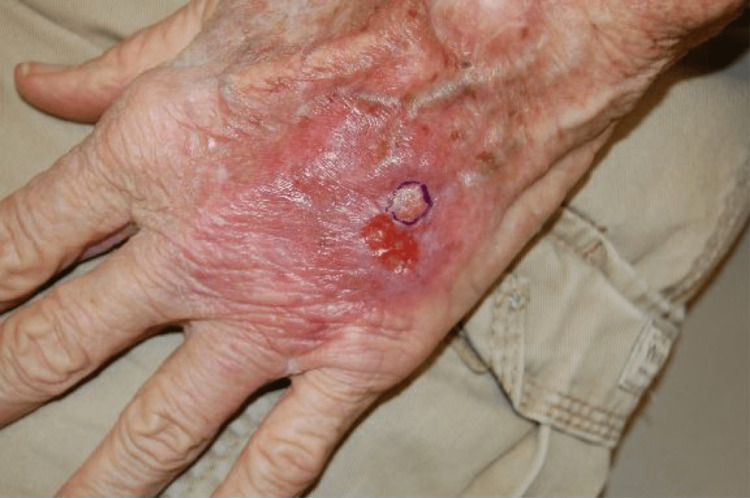
Friable appearing, reddish yellow plaque on an erythematous base on the dorsum of the left hand.

A 78-year-old male with a past medical history of cat scratch disease several years ago presented to dermatology with a yellowish-pink ovoid plaque on his left dorsolateral hand that had been present for about three months (Figure 1). The plaque was 1.7 cm large with some surrounding small, pink papules. Axillary lymph nodes were not assessed at the time of evaluation.

**Figure 2:**
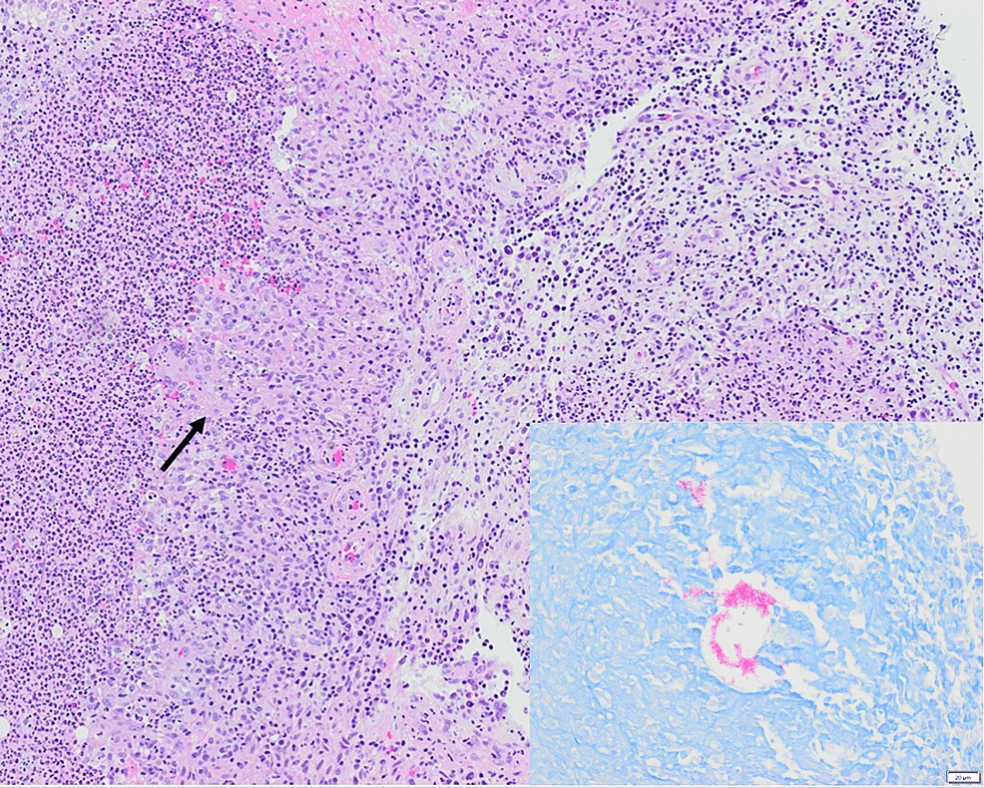
H&E staining revealing loose granulomas adjacent to dense collections of neutrophils. Inset of AFB stain revealing AFB. H&E, hematoxylin and eosin; AFB, acid-fast bacilli

Approximately one to two months prior to developing the plaque on his left dorsolateral hand, the patient had sustained a series of full-thickness scratch wounds on his left hand and wrist from a stray cat. After completing a course of prophylactic amoxicillin-clavulanate, he resumed the application of topical steroids on his left dorsal hand out of concern for pyoderma gangrenosum (PG) formation in the area of the scratch wounds; however, the efficacy of topical steroids for prevention of PG has not been shown in the literature. He had been following with wound care for the past year for treatment of PG and previously had a PG ulcer medial to the new plaque, which resolved with the use of topical steroids, alternating between triamcinolone 0.1% cream and clobetasol 0.05% cream. When the new plaque began to develop, it was initially thought to be recurrent PG given his history, but since the plaque began to worsen despite the use of topical steroids, the patient was referred to dermatology. To rule out a neutrophilic dermatosis, the patient underwent a shave biopsy, which revealed diffuse suppurative and granulomatous dermatitis with associated AFB (Figure 2).

**Figure 3:**
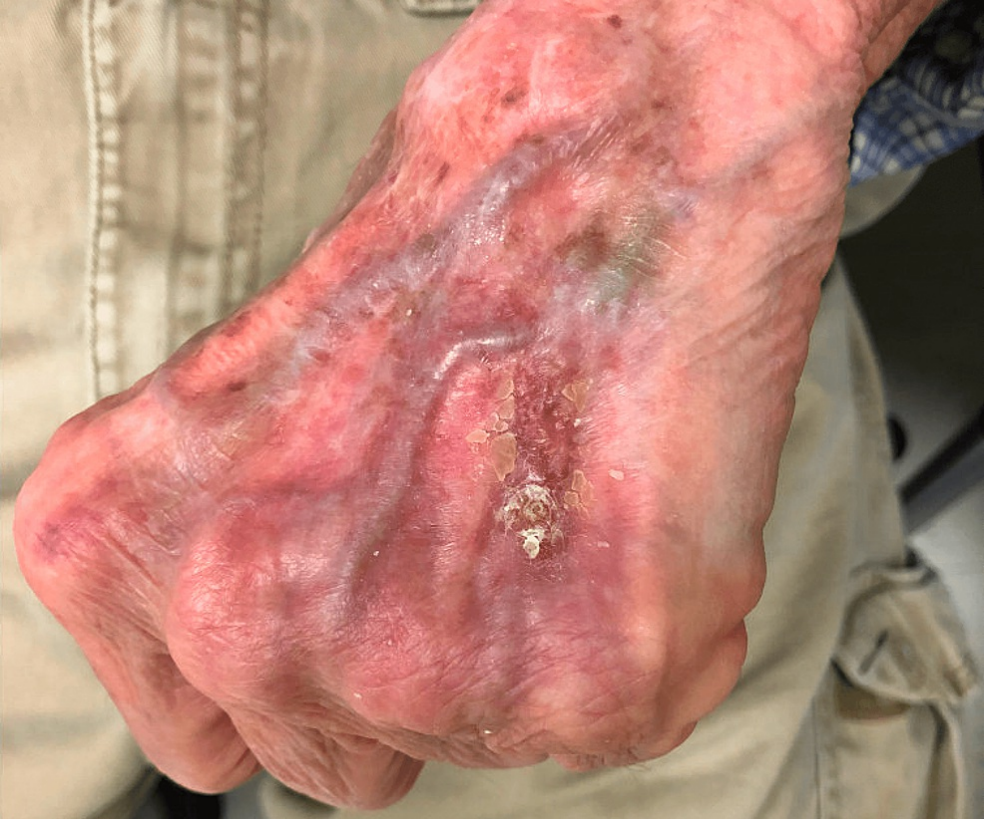
Left dorsal hand with almost complete resolution of initial plaque toward the end of the treatment course with moxifloxacin and clarithromycin.

The patient was instructed to discontinue the use of clobetasol 0.05% cream at that time. Due to these findings, a second biopsy of the lesion was performed and sent for AFB culture, acid-fast smear, aerobic/anaerobic culture, Gram stain, fungal culture, and fungal smear. Routine aerobic culture revealed methicillin-susceptible *Staphylococcus aureus* (MSSA), prompting the initiation of a course of doxycycline. The lesion seemed to grow in size and ulcerate despite the use of doxycycline, so the patient was empirically treated for a cutaneous mycobacterial infection with azithromycin and minocycline. The AFB culture subsequently grew *M. chelonae*, and the patient was ultimately placed on a six-month regimen of moxifloxacin and clarithromycin with an excellent response (Figure 3).

## Discussion

Skin infections with *M. chelonae* may present as cutaneous ulcerative lesions, subcutaneous nodules, and draining fistulas [6]. Patients who have apparent skin infections refractory to treatment for typical aerobic pyogenes (usually *S. aureus* and beta-hemolytic streptococci) or who are immunocompromised should have mycobacterial disease ruled out [7].

To diagnose NTM infection, an acid-fast smear and AFB culture are performed. Identification of Mycobacterium species from skin biopsy culture is the diagnostic standard [4]. Histopathological exam of a suspected cutaneous NTM infection typically shows suppurative granulomas and acid-fast stain may show AFB [8, 9]. At times, initial acid-fast stain and culture can yield false negative results due to a low number of organisms [10].

It has been noted that cats can be reservoirs for NTM pathogens due to the close proximity of owners and their pets in the home environment. Cases of NTM disease related to cat scratches and bites have been reported in the literature, including a skin infection of the forearm by *M. fortuitum* following a cat bite and a skin infection of the face involving *M. marinum* after a cat scratch [10, 11]. Another case involved sporotrichoid-like cutaneous lesions and infection with *M. chelonae* related to cat scratches in the setting of long-term corticosteroid use [12]. Hand infections due to NTM have been reported with some regularity, and the most common causative organism is *M. marinum* [13, 14]. Our patient’s presentation was interesting because he had been applying topical corticosteroids to his left hand for an extended period, which would have caused local immunosuppression and increased susceptibility to infection with *M. chelonae*. While it is impossible to prove that this patient’s NTM infection resulted from the cat scratches, those were the only known potential sources of inoculation recognized.

Treatment of NTM infections is challenging, since these mycobacterial species do not respond to many of the standard medications used to treat tuberculosis and may be multi-drug resistant. In many cases, tetracyclines, macrolides, and fluoroquinolones have been of benefit [4]. Treatment duration and drug regimen vary depending on the organism and site/severity of infection [14].

## Conclusions

This report describes an NTM infection likely transmitted via cat scratch in the setting of topical steroid use. Although cat scratches are more commonly associated with infections due to* S. aureus *or *Bartonella henselae*, cats can serve as reservoirs for NTM, and cutaneous NTM disease should be considered in cases of persistent skin and soft tissue infection after a cat scratch, especially in an immunocompromised host. This includes, as in our case, those patients with localized cutaneous immune deficits due to the use of topical immunosuppressive agents.
